# Changes in malaria morbidity and mortality in Mpumalanga Province, South Africa (2001- 2009): a retrospective study

**DOI:** 10.1186/1475-2875-11-19

**Published:** 2012-01-13

**Authors:** Lindokuhle Ngomane, Christiaan de Jager

**Affiliations:** 1 University of Pretoria Centre for Sustainable Malaria Control, School of Health Systems and Public Health, Faculty of Health Sciences, Pretoria, South Africa

**Keywords:** Malaria, Morbidity, Mortality, Incidence rate, Case fatality rate, Vector control, Indoor residual spraying, Climate

## Abstract

**Background:**

Malaria remains a serious epidemic threat in Mpumalanga Province. In order to appropriately target interventions to achieve substantial reduction in the burden of malaria and ultimately eliminate the disease, there is a need to track progress of malaria control efforts by assessing the time trends and evaluating the impact of current control interventions. This study aimed to assess the changes in the burden of malaria in Mpumalanga Province during the past eight malaria seasons (2001/02 to 2008/09) and whether indoor residual spraying (IRS) and climate variability had an effect on these changes.

**Methods:**

This is a descriptive retrospective study based on the analysis of secondary malaria surveillance data (cases and deaths) in Mpumalanga Province. Data were extracted from the Integrated Malaria Information System. Time series model (Autoregressive Integrated Moving Average) was used to assess the association between climate and malaria.

**Results:**

Within the study period, a total of 35,191 cases and 164 deaths due to malaria were notified in Mpumalanga Province. There was a significant decrease in the incidence of malaria from 385 in 2001/02 to 50 cases per 100,000 population in 2008/09 (*P *< 0.005). The incidence and case fatality (CFR) rates for the study period were 134 cases per 100,000 and 0.54%, respectively. Mortality due to malaria was lower in infants and children (CFR < 0.5%) and higher in those >65 years, with the mean CFR of 2.1% as compared to the national target of 0.5%. A distinct seasonal transmission pattern was found to be significantly related to changes in rainfall patterns (*P *= 0.007). A notable decline in malaria case notification was observed following apparent scale-up of IRS coverage from 2006/07 to 2008/09 malaria seasons.

**Conclusions:**

Mpumalanga Province has achieved the goal of reducing malaria morbidity and mortality by over 70%, partly as a result of scale-up of IRS intervention in combination with other control strategies. These results highlight the need to continue with IRS together with other control strategies until interruption in local malaria transmission is completely achieved. However, the goal to eliminate malaria as a public health problem requires efforts to be directed towards the control of imported malaria cases; development of strategies to interrupt local transmission; and maintaining high quality surveillance and reporting system.

## Background

Malaria has plagued mankind throughout history and still remains one of the major challenges to global health in terms of morbidity, mortality and economic under development [1,2]. According to the World Malaria Report 2010, the global prevalence of the disease was estimated at 225 million cases and 781 000 deaths in 2009 [3]. More than 80% of these cases are estimated to occur in sub-Saharan Africa, especially in remote rural areas with poor access to health services [4,5]. In 2000, malaria was estimated to contribute to the loss of nearly 45 million disability-adjusted life years (DALYs), which represents about 13% of all infectious diseases [6].

South Africa is not exempt from the impact of seasonal and unstable malaria transmission, particularly in the northern and eastern parts of the country [7]. Mpumalanga Province is one of the country's provinces that still experiences unstable malaria transmission, by contributing 44% of the country's notified malaria cases [8]. Due to low transmission levels, immunity to malaria is not thought to exist and infected individuals are therefore prone to severe disease. Between 1987 and 1999 the number of annual malaria cases reported in Mpumalanga Province increased significantly from 1,206 to 11,171 cases [9]. In 2000, the province was severely affected by malaria epidemic due to floods with cases reaching 13,856 [8]. For more than five decades, Mpumalanga Province has maintained a successful control programme, with control strategies including rapid detection and treatment of confirmed malaria cases at Primary Health Care clinics and vector control through IRS with insecticides and focal larviciding [9]. *Plasmodium falciparum *accounts for the majority of the cases, transmitted mainly by *Anopheles arabiensis *[10].

Large-scale malaria control operations based on house-spraying with DDT (dichlorodiphenyltrichloroethane) were initiated in South Africa during the 1940s leading to a decline in the level of malaria transmission in large parts of the country and elimination of the major malaria vectors *Anopheles funestus *and *Anopheles gambiae *[11]. However, following several identified environmental concerns and social resistance (re-plastering over DDT and refusing household access) due to the increased incidence of bedbugs associated with the use of DDT [12], malarious provinces including Mpumalanga discontinued the use of DDT in favour of synthetic pyrethroid insecticides in 1996 [13].

The discontinuation of DDT coincided with a sudden reappearance of the malaria vector *An. funestus *in KwaZulu-Natal [14] and an upsurge in malaria cases [15]. In the absence of data exploring the relationship between the high incidence of malaria and the use of DDT during that period, several possible factors such as climate change, vector biology and behaviour, drug and insecticide resistance and flawed insecticide application were put forward in an attempt to quantify the underlying reasons for the increase [16]. In view of these trends, South Africa had to revert to DDT as an insecticide of choice for IRS and change the first-line treatment from sulphadoxine-pyrimethamine (SP) to Coartem^® ^[17].

Although malaria vector control through IRS in South Africa has proved to be successful in reducing malaria transmission [13], in Mpumalanga Province significant gaps remain in terms of its direct impact on malaria mortality and morbidity reduction over the years. There have not been any studies previously conducted to assess the impact of vector control interventions on the burden of malaria using the provincial epidemiological data. The importance of analysis of data on the prevalence of disease in relation to expanded control interventions is well documented in other malaria endemic areas [18]. Understanding the relationship between climate, control methods and malaria has been shown to assist in providing early warning in malaria increases or potential outbreaks as well as in improving the control programme [19,20].

The present study aimed to assess the changes in malaria morbidity and mortality during the past eight malaria seasons in Mpumalanga Province, while taking into account the potential effect of factors such as climate and IRS, which might have influenced these changes.

## Methods

### Study area

**Figure 1:**
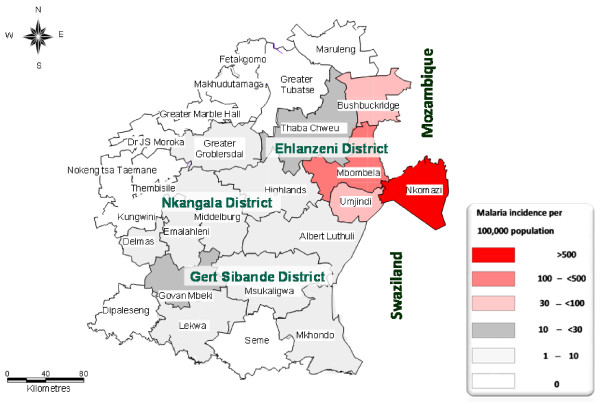
**A map of Mpumalanga Province, South Africa**. The map depicts the spatial distribution of the incidence of malaria by district and municipal area (2001-2009).

Mpumalanga Province is administratively divided into three districts: Ehlanzeni, Nkangala and Gert Sibande. The districts are further sub-divided into 24 municipalities (Figure 1). Vector control strategies in Mpumalanga Province include regular spraying of interior walls of houses carried out in the high risk malaria areas, a seasonal round from August to February each year. The residual insecticides of choice for indoor application are DDT for traditional structures and synthetic pyrethroids and carbamates for western-type structures. To control mosquito larvae, larviciding operation using temephos (organophosphate) is carried out on identified breeding sites.

### Data collection

All malaria records were obtained from the provincial Integrated Malaria Information System (IMIS) under Malaria Control Programme of the Department of Health. Malaria morbidity and mortality data consisting of both passive and active cases based on definitive diagnosis reported from 2001 to 2009 were extracted from the IMIS. The data consisted of the following variables: date of diagnosis, age, gender, facility name, municipality, source country, province and locality. Since malaria transmission in South Africa is seasonal, data were aggregated by season rather than by calendar year. A malaria season was defined to be the period from the beginning of July to the end of June the following year. Data for the Bushbuckridge municipality (Figure 1), which formerly belonged to Limpopo Province (Bohlabela district), were only captured on the Mpumalanga IMIS from 2006, following its integration into Ehlanzeni district [21].

IRS data were obtained from the computerized spraying management system, which is maintained in Mpumalanga Province. Activities pertaining to IRS were reported per spray season, which takes place from August to February the following year. Variables recorded in this database include: the type of insecticide applied, structures targeted for spraying, number of structures sprayed, amount of insecticide used and localities sprayed.

Climate records for 2001 to 2009 were obtained from the South African Weather Service and comprised of the following variables: mean monthly temperature (°C) (minimum and maximum), relative humidity (%) and rainfall (mm).

### Data analysis

Data were entered into Microsoft Excel and Access then converted into Stata (version 11) for statistical analysis [22]. Descriptive statistics were computed, presenting the frequencies, proportions with their 95% confidence intervals (CIs), mean values and standard deviations (SD). Malaria incidence rates were estimated using the mid-year population estimates for the years 2001 to 2009 [23] as well as population counts from the 2001 national census [24]. The incidence of malaria per 100,000 population were computed per malaria season by age, gender and geographical area-specific (country, province, district and municipality). Malaria case fatality rates (CFRs) for the period under review were computed to evaluate the quality of case management for severe and complicated malaria. Pearson's chi square tests were used to evaluate the associations between categorical variables (gender, country, province, district and municipality) and malaria outcomes. The chi square test for trend was used to test for trends over the eight malaria seasons. The effect of IRS intervention on malaria incidence was quantified by computing descriptive statistics of the number of structures sprayed and amount of insecticide applied in relation to malaria cases notified per spray season.

Time series analysis was used to assess the effect of climatic factors on malaria transmission by fitting ARIMA (Autoregressive Integrated Moving Average) models on the data for 2001/02-2008/09. The models were developed using the mean monthly malaria case numbers as dependent variable and mean monthly climatic factors as independent variables. The goodness-of-fit of the models were checked for adequacy using appropriate diagnostic methods (i.e. plotting the residuals of the model). Differences at *P *< 0.05 were regarded as statistically significant.

This study was approved by the Faculty of Health Sciences Ethical Committee at the University of Pretoria (Ref No. 72/2010: 28/04/2010) and the Department of Health in Mpumalanga Province (Ref No. 3102: 25/06/2010).

## Results

### Malaria case notification and incidence rate

From July 2001 to June 2009, a total of 35,191 (mean 4,399; 95% CI: 1,631 - 7,167) confirmed malaria cases were notified in Mpumalanga Province (Table 1). The number of cases per malaria season ranged from 12,125 cases in 2001/02 to 1,805 cases in 2008/09, with the seasonal mean number of reported cases ranging from 1,010 to 150. Over the whole study period, there has been a steady decline in malaria cases with minor biennial fluctuations. A steep reduction of almost 70% was observed between 2001/02 and 2002/03 followed by notable peaks in 2003/04 (4,710), 2005/06 (4,680) and in 2007/08 (2,421).

**Table 1:** Reported malaria cases and malaria-attributed deaths, Mpumalanga Province, 2001/02 -2008/09 malaria seasons

Malaria season	No. of reported malaria cases (Proportion -%)	Mean reported cases per season (SD)	Malaria incidence rate	Malaria attributed deaths	CFR (%)
2001/02	12125 (34)	1010 (599.6)	385	34	0.28

2002/03	4050 (12)	338 (192.8)	126	14	0.35

2003/04	4710 (13)	393 (197.6)	145	30	0.64

2004/05	3112 (9)	259 (137.2)	96	18	0.58

2005/06	4680 (13)	390 (361.9)	139	25	0.53

2006/07	2288 (7)	191 (90.7)	65	16	0.71

2007/08	2421 (7)	202 (166.5)	68	18	0.74

2008/09	1805 (5)	150 (94.9)	50	9	0.51

Incidence rate per 100,000 population

SD: Standard deviation; CI: Confidence interval; CFR: Case fatality rate

The overall incidence of malaria for Mpumalanga Province in 2001-2009 was 134 cases per 100,000 population (95% CI: 44.4 - 224.1), ranging from 385 in 2001/02 to 50 cases per 100,000 in 2008/09, indicating a significant decrease over time (*x*^*2 *^= 21.3; *P *= 0.003).

### Malaria according to age and gender

The incidence estimates of malaria according to age are given in Table 2, indicating that all age groups were affected. The mean age was 26 years (16 SD) and range (0-96 years). Of all the notified cases, 9% (95% CI: 8.8%-9.4%) were among those under the age of five years, 14% (95% CI:13.8% - 14.5%) among those five-14 years, 24% (95% CI: 23.6% - 24.5%) in those in the age group 15-24 years, 26% (95% CI: 25.8% - 24.5%) in the 35-44 years age group, 15% (95% CI: 14.3% - 15%) for age group 35-44 years and those over the age of 45 years made up the difference.

**Table 2:** Age and sex-specific malaria cases, incidence and case fatality rate, Mpumalanga Province, 2001/02 - 2008/09 malaria seasons

Age group (years) and sex	No. of reported cases (Proportion -%)	Mean reported cases per season(SD)	Malaria incidence rate	Malaria-attributed deaths	CFR (%)
0-4	3209 (9)	401(321.8)	97	9	0.28

5-14	4979 (14)	622 (624.1)	70	7	0.14

15-24	8450 (24)	1056 (894.8)	128	19	0.22

25-34	9255 (26)	1157 (720.1)	190	36	0.39

35-44	5158 (15)	645 (413.9)	151	36	0.70

45-54	2453 (7)	307 (188.0)	106	30	1.22

55-64	1072 (3)	134 (108.7)	75	14	1.31

>65	615 (2)	77 (67.4)	50	13	2.11

Male	20855 (59)	2607 (1800)	167	85	0.41

Female	14336 (41)	1792 (1517)	106	79	0.55

Incidence rate per 100,000 population

SD: Standard deviation; CI: Confidence interval; CFR: Case fatality rate

Males had a higher risk of contracting malaria than females in all age groups, accounting for 59.3% of confirmed cases compared to 40.7% in females; this trend was statistically significant (*P *< 0.001). The incidence rate was 167 cases per 100,000 in males (95% CI: 164.6 - 169.2) versus 106 cases per 100,000 in females (95% CI: 104.7 - 108.1) (Table 2).

### Malaria-attributed mortality and case fatality rate

A total of 164 deaths attributed to malaria were recorded between 2001 and 2009. The annual number of malaria deaths had significantly declined by approximately 74%, from 34 in 2001/02 to 9 in 2008/09 (*x*^2 ^= 28.2; *P *< 0.001). The case fatality rate fluctuated over the years ranging from 0.28% to 0.74%, higher during the 2006/07 (0.71%) and 2007/08 (0.74%) malaria seasons and subsequently followed by a marked decline in the last malaria season (2008/09) reaching the 0.5% national target for malaria case fatality rate in South Africa (Table 1).

Deaths related to malaria increased with increasing age, more pronounced in the age group 25- 34 years in females and 35-44 years in males (Table 2). The data shows that malaria mortality was lower in infants and children (under five and five-14 years), accounted for only 9% of all deaths attributed to malaria. However, malaria deaths increases from the age group 15-24 years (12%), reaching a peak at the age group 25-34 and 35-44 years (both groups comprised 22%) and the remaining age groups (>45 years) combined, constituted 35%. There were significant differences in the case fatality rate among males and females (*x*^*2 *^= 21.3; *P *< 0.001) with mean CFR of 0.41% and 0.55%, respectively. The severe impact of complicated malaria was observed in the older age groups from age 45-54 years to those above the age of 65 years, the CFR ranged from 1.31% to 2.11%.

### Seasonal malaria variation

**Figure 2:**
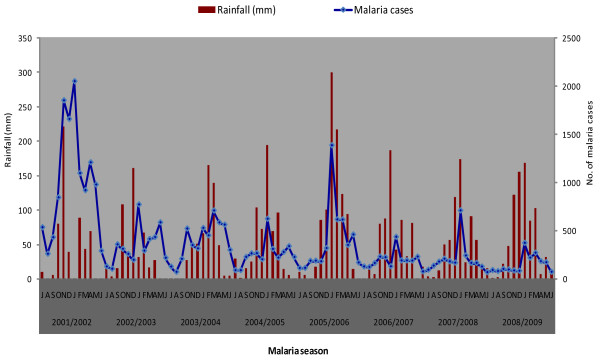
**Relationship between monthly malaria transmission and rainfall, Mpumalanga Province, 2001/02 -2008/09 malaria seasons**.

Although malaria cases were prevalent throughout each year, transmission was distinctly seasonal, increasing from September to May and decreasing thereafter. Peak transmission occurred from December to February each year (Figure 2). The pattern of malaria transmission was more pronounced in the 2001/02 malaria season. In general, there was a downward temporal trend with some inter-annual variation throughout the nine-year period.

### Geographical sources of malaria infection

About half (50.1%) of the notified malaria cases were acquired in Mpumalanga Province. The remainder of the cases (49.8%) were imported from other regions, while <1% (37) of these, the source of infection was not captured. The distribution of malaria varied widely across the three districts of Mpumalanga Province (*x*^2 ^= 43.4; *P *< 0.001) (Figure 1). A large proportion of the province's cases were notified in Ehlanzeni district, accounting 96.5% (95% CI: 96.3% - 96.7%) of all the notified cases. The other two districts, Gert Sibande and Nkangala notified the lowest number of cases during the past eight malaria seasons, contributed 0.7% (95% CI: 0.7% - 0.8%) and 0.4% (95% CI: 0.4% - 0.5%) respectively.

Ehlanzeni district is sub-divided into five municipalities; Nkomazi, Mbombela, Umjindi, Bushbuckridge and Thaba Chweu. The majority of malaria cases were notified in Nkomazi municipality accounting for 73% of all the notified cases followed by Mbombela (18%), Umjindi (3%), Bushbuckridge (3%) and Thaba Chweu (1%). The Kruger National Park and surrounding lodges were included under Ehlanzeni district and accounted for 2% of all the cases.

The spatial distribution of the incidence of malaria in Mpumalanga Province indicates that Ehlanzeni district is mostly affected by seasonal malaria (Figure 1). In particular, Nkomazi municipality remains the high-risk area with an incidence rate of more than 500 cases per 100,000. Of these 52.5% (12,574) were local cases while 47.5% (11,391) imported cases (originating from other countries). The low risk areas are mainly Gert Sibande and Nkangala districts, where the incidence rates are less than 10 per 100,000 population.

### Effect of climate on malaria

Climatic conditions remained relatively stable throughout the study period, apart from the rainfall anomaly recorded in 2005/06, which was 30% higher (968 mm) than the annual average (650 mm) for Mpumalanga Province. During this period, malaria cases were higher than the previous season as well as the subsequent seasons. The relationship between rainfall and malaria is illustrated in Figure 2. Depending on the amount of rainfall, upsurges in malaria transmission are seen with a time lag of one to two months. Furthermore, the ARIMA model showed that rainfall was the only climatic variable significantly associated with the transmission of malaria in Mpumalanga Province (*P *= 0.007). There was no significant association between temperature and relative humidity and monthly malaria cases (*P *> 0.05) (Table 3).

**Table 3:** Time series analysis (ARIMA model) of the incidence of malaria on climatic variables in Mpumalanga Province

Variables	Coefficient	Standard error	*P*-value
Rainfall	-0.062	0.023	0.007

Tmin	0.016	0.055	0.768

Tmax	0.012	0.027	0.668

RH	0.006	0.012	0.585

Constant	-0.227	0.006	0.000

Tmin: Minimum temperature; Tmax: Maximum temperature; RH: Relative humidity

### Effect of IRS intervention

Table 4 presents the trends in IRS during the past eight malaria seasons (2001/02-2008/09). The table gives a summary of the amount of residual insecticide applied, the number of structures covered with IRS and spray coverage achieved. The data shows that a total of 406,413 kg (406 tonnes) of the different residual insecticides (DDT, K-Othrine, Baythroid and Fendona) was used to spray a total of 2,865,592 structures during the study period. Over half of the structures were sprayed with DDT (54%) and the other insecticides made up the difference. The spray coverage for the eight malaria seasons was 85.4% (95% CI 83.2% - 87.6%) ranging from 44% to 100%.

**Table 4:** Descriptive statistics of residual insecticides applied and the number of structures sprayed in Mpumalanga Province during 2001/02 - 2008/09 malaria seasons

Insecticide	Amount insecticide applied (kg)	Mean insecticide applied (SD)	Number structures sprayed	Mean structures sprayed (SD)	Spray coverage (per malaria season)
Baythroid	75	37.3 (52.7)	7517	3758.5 (5311.1)	83.5%

DDT	176571	5044.9 (5409.4)	1588586	45388.2 (50742.9)	81.7%

Fendona	220912	31558.9 (38817)	280341	40048.7 (49368.5)	88.4%

K-Othrine WP	6312	263 (338.1)	322797	13449.9 (17070.1)	85.1%

K-Othrine WG	2543	106(141.6)	666351	27764.6 (38398.1)	90.5%

Total	406413	4417.5(13304.4)	2865592	31148(41899)	85.4%

WP: Wettable powder; WG: Wettable granules; Kg: Kilogram; SD: standard deviation

**Figure 3:**
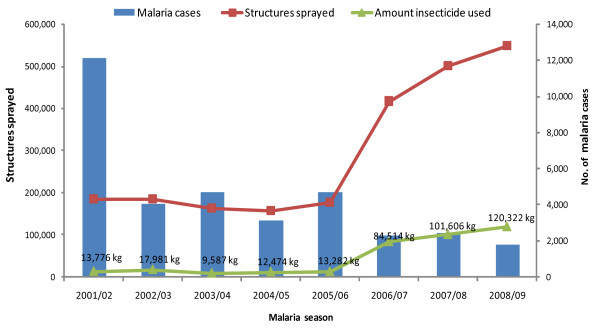
**IRS intervention in relation to malaria cases notified by season in Mpumalanga Province, 2001/02 - 2008/09 malaria seasons**. The figure presents data on total structures sprayed and the total amount of insecticide applied in relation to malaria cases in Mpumalanga Province.

The direct link between expanded IRS in relation to malaria cases is illustrated in Figure 3. Spray coverage and insecticide application remained stable during the first four malaria seasons (2001/02 to 2004/05), however, an increasing trend was observed from 2006/07 to 2008/09. Changes in notified malaria cases were related to the scale-up of IRS activities, showing a rapid decreasing trend with small peaks, during the same time period.

## Discussion

The time trends shows a gradual decline in malaria morbidity and mortality in Mpumalanga Province over the past eight malaria seasons, representing an 85% reduction in the annual number of confirmed malaria cases and 74% in the number of deaths-attributed to malaria. A previous study, estimating the burden of malaria in Mpumalanga Province between 1987 and 1999, showed fluctuating trends coupled with flood-related malaria epidemics in 1996 and 2000 [9]. Since 2002, the province has seen a marked decline in malaria cases and deaths, with no epidemics detected. The declining trend in malaria incidence demonstrated in this study is consistent with previously published results from other malarious provinces of South Africa; KwaZulu-Natal [25] and Limpopo [26].

Notable peaks in the number of notified cases were observed during the 2001/02, 2003/04 and 2005/06 malaria seasons. The 2001/02 peak was followed by a steep reduction of almost 70% in the subsequent season. This could be a result of the change in drug policy to combat parasite resistance to SP [27]; the re-introduction of DDT; and the Lubombo Spatial Development Initiative (LSDI), a joint development programme implemented to control malaria in Mozambique and Swaziland [28]. The introduction of artemisinin combination therapy (ACT) for treatment of uncomplicated malaria in 2003 and 2004 [27] could partly explain the upsurge in malaria cases during the 2003/04 malaria season. However, it is possible that drug policy was not the only change that was introduced during this period. Further research is required to explain this scenario. The 2005/06 pattern may be attributed to the integration of Bushbuckridge municipality into Mpumalanga Province; the abandonment of the requirement of entry visa between South Africa and Mozambique [29] leading to large population movement between the countries thus bringing about importation of malaria cases; and rainfall anomaly.

In this present study, the finding of peak malaria incidence in the young adult age group (15-44 years) may be related to outdoor behavioural risk factors such as leisure patterns and sleeping arrangements leading to exposure to infective mosquito bites [30]. The low proportion of cases in infants and children further supports possible predominance of outdoor transmission since small children tend to spend more time indoors particularly during mosquito biting time. In Tanzania, it was found that high usage of intra-domiciliary vector control tools may have altered vector feeding patterns from indoor to outdoor transmission, suggesting the need for additional vector control tools that target outdoor biting mosquitoes, such repellents and larval control [31].

This study found significant differences (*P *< 0.001) in malaria incidence between males and females. This was similar to previous observations in Mpumalanga [9], KwaZulu-Natal [32] and Limpopo [26] Provinces. In some societies, men have a much greater risk of contracting malaria due to occupational reasons, particularly those that work in mines, fields or migrate to areas of high endemicity for work [33]. The study conducted in Ethiopia provides evidence of the relationship between occupation and malaria risk; the authors found that highland migrant labourers were vulnerable to malaria while migrating to find agricultural work [34]. A similar scenario may exist in Mpumalanga Province.

Analysis of the findings reveals that the burden of malaria in Mpumalanga Province is strongly connected to importation of malaria parasites by population movement as almost half (48%) of the cases reported in Mpumalanga Province were acquired in Mozambique. Trend analysis of these cases reveals that 74% were among males between the ages of 15 and 44 years; confirming that the majority of the imported cases were introduced by young adult males crossing the border to seek work opportunities in South Africa. In view of the high proportion of cases acquired in Mozambique, intensification of the regional cross-border and intersectoral collaboration approach is vital in order to lower the risk of re-importation of malaria infections.

Malaria transmission in Mpumalanga Province shows inter-annual variation from 2001 to 2009 with a distinct malaria transmission season, prominent peaks in January and February. The reasons for the January/February peaks could be attributed to favourable climatic conditions for malaria transmission (peak summer season) or introduced parasites by human immigration from various places of origin following the December holiday season. This finding suggests important implications for the control programme in terms of timing when directing efforts in controlling malaria.

Although a large proportion of the cases reported in Mpumalanga Province were imported, the province still accounted for half (50.1%) of the total cases notified, which indicates the recurrence of local malaria transmission. The incidence of malaria was found to be most pronounced in Ehlanzeni district (low altitude region) than in the high altitude districts (Nkangala and Gert Sibande), suggesting the effect of altitude on malaria transmission. The burden of the disease in the province lies in Nkomazi municipality by contributing 73% of the province's malaria cases. Malaria transmission in this area can be attributed to factors such as its sub-tropical weather conditions, close proximity to high malaria transmission parts of Mozambique as well as intensive agricultural practices. The link between malaria and agriculture has a long history, in particular irrigation, by creating suitable vector breeding sites and facilitating malaria transmission.

Malaria case fatality rates fluctuated over the entire study period. The overall CFR was 0.54% which is not much significantly higher than the national target. However, it is important for the province to further decrease the CFR to below 0.5% through improved case management. Unlike other African countries, where malaria is a major cause of infant and child mortality, in Mpumalanga Province malaria-attributed mortality was lower in infants and children. Severe illness due to malaria was higher among adults, with the CFR reaching 2.1% in those over the age of 65 years compared to 0.28% and 0.14% for under fives and five-14 age groups. The enquiry into all deaths-attributed to malaria in Mpumalanga Province in 1999, revealed that late presentation to health care facilities was strongly associated with increased mortality due to malaria [35]. In another study in South Africa, it was found that co-morbid diseases, especially HIV co-infection and poor management of malaria-related complications led to mortality outcomes [36]. This suggest the need to maintain sustainable training programmes for all health care workers in all levels of health facilities in both low and high risk transmission areas as well as community health promotion and education.

It can be reported that rainfall has played a significant role in the transmission of malaria in Mpumalanga Province. The transmission season followed a distinct rainfall pattern and fluctuated considerably from year to year according to rainfall variability, with heavy rainfall associated with increased number of reported cases. Similar results were reported in KwaZulu-Natal where they observed a direct and predictable relation between rainfall and malaria transmission [37].

It has been suggested that anthropogenic climate change is expected to directly affect the behaviour and geographical distribution of mosquitoes and the life cycle of the parasite and thus changing the epidemiology of the disease [38,39]. Casman et al. [40], however notes that since climate can correlate with transmission intensity, it can greatly affect the success or failure of control and eradication programmes. The authors however point out that in fringe transmission areas like South Africa, malaria surveillance and control maybe sufficient to mitigate any increases in transmission brought about by climate change. Mpumalanga Province needs to maintain high quality surveillance system to facilitate immediate detection, notification and response to outbreaks that may be triggered by climate anomalies.

The elimination of malaria transmission in some temperate regions of the world during the eradication era in the 1950s to 60s was largely based on IRS, which illustrates its programmatic effectiveness in malaria control [41]. Numerous studies have shown that intensive IRS campaigns have substantially reduced levels of infection and incidence of malaria [42]. In some settings it has been reported that the IRS intervention was associated with marked decreases in malaria transmission by more than 50% [43].

A study conducted in Uganda to assess the impact of IRS on malaria morbidity after a single round of spraying with lambda-cyhalothrin found a consistent decrease in the number of patients diagnosed with clinical malaria in the first four months after IRS [44]. In South Africa, marked reductions in the number of confirmed cases and deaths in Mpumalanga and KwaZulu-Natal Provinces were observed following the introduction of IRS campaigns in Mozambique and Swaziland through the LSDI. In KwaZulu-Natal Province, a significant reduction in the number of cases in most endemic areas of the province was reported following the re-introduction of DDT for vector control [45].

Although increased IRS coverage has significantly contributed to the visible decreasing trends in malaria morbidity and mortality, other factors such as the combination of preventative interventions (early diagnosis, case management and effective treatment with ACT), low numbers of mosquito vectors, improved education and awareness, and improving socio-economic indices, had played a role in the marked reduction in the burden of malaria in Mpumalanga Province.

Although IRS has had beneficial effects in the history of malaria control and prevention, there is increasing awareness and concerns from new scientific evidence regarding the safety of insecticides (i.e. DDT) on humans and the environment [42]. More recently, the Pine River Statement revealed substantial evidence that DDT and DDE pose a serious risk to human health, particularly due to IRS for malaria vector control [46]. Other studies conducted in South Africa reported several risks associated with non-occupational exposure, such as male reproductive effects [47,48]. In view of these public health concerns, efforts needs to be directed towards the development of new tools for malaria vector control in order to minimize adverse health effects and halt endemic transmission.

Based on the findings of this study, the Mpumalanga Malaria Control Programme needs to consider the following in order to achieve the goal to eliminate malaria; (i) address importation of malaria cases through intensification of the regional cross-border collaboration efforts; (ii) develop strategies to interrupt local transmission and transmission risk through identification of asymptomatic infections and effective treatment of all infections before transmission can occur and; (iii) maintain strong surveillance and reporting systems through vigilant monitoring of the data collection (i.e. completion of notification forms) and capturing process in order to maintain a consistent information system which is a cornerstone of a successful malaria elimination programme.

It is acknowledged that this study has several limitations given that it relies mainly on routine surveillance data. Firstly, the problem with surveillance data is that the quality might be subjected to reporting inconsistencies and incompleteness, emanating from lack of systematic inclusion of data from other sources such as traditional healers, faith-based organizations and self-treatment cases. Secondly, over-reporting of malaria cases could have also occurred in the high-risk areas due to high awareness and advocacy regarding malaria among health care workers. Finally, it is possible that some confounding factors that may have influenced the changes in the burden of malaria were not addressed in this study, such as the effect of other preventive interventions, changes in vector population and socio-economic status.

Despite these limitations it can be noted that routinely collected data through the provincial surveillance system remains the basis for measuring malaria trends over time. The main advantage of the surveillance data is that it includes asymptomatic cases through passive case detection, thus including cases which might have gone unreported in health care facilities. In view of this, the surveillance system data has captured the picture of malaria transmission patterns in Mpumalanga Province and these findings can be used to strengthen malaria control efforts.

## Conclusions

The findings show that Mpumalanga Province has achieved the goal to reduce malaria to low levels through the scale-up of IRS in combination with other control interventions. These results highlight the need to continue with current control strategies until interruption in local malaria transmission is completely achieved and sustainable control strategies implemented. It is clear that a comprehensive intervention plan which encompasses a combined set of widespread interventions is required in order to ensure continuous improvement in malaria control and subsequent elimination.

## Competing interests

The authors declare that they have no competing interests.

## Authors' contributions

LN conceived and designed the study, analysed the data and drafted the manuscript. CDJ was involved in the conception and design of the study, supervised the writing of the manuscript and critically revised the report. All authors read and approved the final manuscript.
